# Puerarin inhibits NHE1 activity by interfering with the p38 pathway and attenuates mitochondrial damage induced by myocardial calcium overload in heart failure rats: Puerarin inhibits NHE1 activity

**DOI:** 10.3724/abbs.2023269

**Published:** 2024-01-26

**Authors:** Guopin Pan, Baoyue Cui, Mingming Han, Laibiao Lin, Yinlan Li, Ling Wang, Shuang Guo, Yaling Yin, Heqin Zhan, Peng Li

**Affiliations:** 1 Sino-UK Joint Laboratory of Brain Function and Injury and Department of Physiology and Neurobiology Henan International Joint Laboratory of Cardiovascular Remodeling and Drug Intervention School of Basic Medical Sciences College of Pharmacy Xinxiang Medical University Xinxiang 453003 China; 2 Hubei Key Laboratory of Diabetes and Angiopathy Hubei University of Science and Technology Xianning 437100 China; 3 Nanyang Second General Hospital Nanyang 473001 China; 4 College of Pharmacy Heilongjiang University of Chinese Medicine Harbin 150040 China

**Keywords:** puerarin, p38 pathway, NHE1, heart failure, TGF-β, proinflammatory cytokines

## Abstract

Previous studies have shown that puerarin plays a key role in protecting humans and animals from cardiovascular diseases. The exact mechanism of the therapeutic effect of puerarin on various cardiovascular diseases (protective effect on cardiomyocytes) is still unclear. In the present study, we identify the role of puerarin in an animal model of experimental heart failure (HF) and explore its underlying mechanisms. The HF rat model is induced by intraperitoneal injection of adriamycin (ADR), and puerarin is administered intragastrically at low, medium, and high concentrations. We demonstrate that puerarin significantly improves myocardial fibrosis and inflammatory infiltration and, as a result, improves cardiac function in ADR-induced HF rats. Mechanistically, we find for the first time that puerarin inhibits overactivated Na ^+^/H ^+^ exchange isoform 1 (NHE1) in HF, which may improve HF by decreasing Na ^+^ and Ca ^2+^ ion concentrations and attenuating mitochondrial damage caused by calcium overload; on the other hand, puerarin inhibits the activation of the p38 pathway in HF, reduces the expressions of TGF-β and proinflammatory cytokines, and suppresses myocardial fibrosis. In conclusion, our results suggest that Puerarin is an effective drug against HF and may play a protective role in the myocardium by inhibiting the activation of p38 and its downstream NHE1.

## Introduction

HF, with high morbidity and mortality, is the outcome of many end-stage heart diseases that result in a heavy health burden [ 1, 2]. Current HF therapy focuses on symptomatic treatment or delays the progression of the disease by reducing heart rate and cardiac preload and afterload [3]. Despite significant innovations in the medical treatment of HF in recent decades, its incidence continues to increase [4], making it worthwhile to understand the underlying pathological changes in HF and to find effective medicines.

The prevention and cure of HF with natural products are applicable and valuable research fields because plant sources are considered to be less toxic, with fewer side effects than synthetic medicines. Puerarin (Pue) is the main bioactive ingredient isolated from the root of the wild legume *Pueraria lobata* (wild) [5] and has been widely studied due to its multiple pharmacological effects. Emerging studies over the past few decades have demonstrated the efficacy of Pue in the prevention and treatment of cardiovascular diseases, including atherosclerosis, hypertension, cardiac hypertrophy, cardiovascular complications of diabetes, myocardial infarction, and HF [6]. The therapeutic effects of Pue on a variety of cardiovascular diseases work through multiple targets and pathways. There is increasing evidence that Pue can modulate Na ^+^ [7], K ^+^[ 8, 9], and Ca ^2+^ [10] channels and regulate the concentration of these ions in cardiomyocytes to exert its anti-cardiac injury effects. However, studies of the effects of Pue on specific ion channels have been performed by using various channel inhibitors, not by directly recording ion currents, and other regulatory mechanisms for changes in the concentrations of these ions cannot be excluded.

Na ^+^/H ^+^ exchange isoform 1 (NHE1) is a ubiquitously expressed housekeeping glycoprotein that plays a key role in maintaining intracellular pH and regulating intracellular sodium and calcium concentrations through the exchange of intracellular H ^+^ and extracellular Na ^+^ [ 11– 13]. Previous reports have suggested that enhanced expression and activity of NHE1 are associated with the development of different cardiac pathologies [14], including cardiomyocyte hypertrophy (CH) [ 15, 16] and ischemia/reperfusion (I/R) injury [17], in various experimental models. The elevated Na ^+^ level in HF [18] may be related to the increase in Na ^+^/H ^+^ exchangers. However, there are no studies on the effect of Pue on NHE1 expression in the HF myocardium. Previously, the ADR-induced rats were used for HF studies. ADR is a potent anti-neoplastic agent employed in a variety of carcinomas. However, the use of ADR has been limited due to its severe cardiotoxic side effect, which causes fatal congestive heart failure [ 19, 20].

In this study, we determine the pharmacological effects of Pue in an ADR-induced rat HF model and further explore the potential cardioprotective mechanisms of Pue by studying its effect on NHE1 in the myocardium.

## Materials and Methods

### Key reagents

ADR was purchased from Duly Biotechnology (Nanjing, China); Pue was purchased from Selleck Chemicals (Houston, USA). p38 MAPK antibody (mouse, Cat No. 66234-1-lg) and NHE1 antibody (mouse, Cat No. 67363-1-lg) were from Proteintech (Wuhan, China); phospho-p38 MAPK antibody (Thr180+Tyr182) (rabbit, Cat No. ab4822), GAPDH antibody (rabbit, Cat No. ab9485), and β-actin antibody (rabbit, Cat No. ab8227) were from Abcam (Cambridge, USA). HRP-goat anti-rabbit recombinant secondary antibody (Cat No: RGAR001) and HRP-goat anti-mouse recombinant secondary antibody (Cat No: RGAM001) were from Proteintech (Wuhan, China).

### Study design

Male Sprague-Dawley rats (8 weeks old, 180‒220 g) were provided by the Experimental Animal Center of Zhengzhou University (Henan Experimental Animal Center). Before the beginning of the experiment, the rats were adaptively fed for one week. All rats were kept individually in cages at a room temperature of 18‒22°C with a 12 h light/dark cycle and allowed free access to food and water. The animal model of HF was induced by ADR [21]. Briefly, rats were injected intraperitoneally with ADR (ADR group) (3 mg/kg) once a week for 6 consecutive weeks with a cumulative dose of 18 mg/kg. A 0.9% sodium chloride solution was given as a vehicle control (control group). For the *in vivo* study, we predetermined the group size and considered sample loss. Animals that died during the experiment were excluded from the study. The predetermined group size was retained by adding replacement animals to the affected group. Rats that received ADR injections and survived were randomly divided into four groups: ADR group, Pue low-dose treatment group (50 mg/kg, Pue-L), Pue middle-dose treatment group (100 mg/kg, Pue-M), and Pue high-dose treatment group (150 mg/kg, Pue-H) ( *n*=10 each group). Pue was dissolved in ddH _2_O to a final concentration of 37.5 mg/mL and administered via gavage every day for 4 weeks. The study was carried out in strict accordance with the recommendations in the Guide for the Care and Use of Laboratory Animals of the National Institutes of Health. The animal experiment protocol was reviewed and approved by the Ethics Committee of Xinxiang Medical University (Xinxiang, China).

### Echocardiography

Echocardiography was performed as described previously [22]. On the final day of the experiment, all rats were anaesthetized with pentobarbital sodium (35 mg/kg intraperitoneally) plus carprofen (5 mg/kg subcutaneously). The echocardiography of all rats was performed with a GE Vivid 7 (GE Health Medical, Milwaukee, USA). Ultrasonography with standard parasternal and apical views was performed in rats in the left lateral recumbent position. Then, high-quality 2-dimensional and M-mode images of the left ventricle from the parasternal short-axis views [23] were obtained. Images were digitized in cine-loop format and stored. Left ventricular (LV) ejection fraction (EF%) and stroke volume (SV) were taken as measurements of LV systolic function. All echocardiography was performed by the same investigator who was blinded to the treatments.

### Myocyte preparation

Adult rat ventricular myocyte isolation was performed using a previously reported method with some modifications [24]. Briefly, hearts were removed from rats anaesthetized with sodium pentobarbital (60 mg/kg intraperitoneal) and immediately attached to an aortic cannula. The heart was first perfused with CaCl _2_-free modified Tyrode’s solution for 5 min and digested with 0.3 mg/mL collagenase (Type 2, Worthington Biochemical, Freehold, USA), 0.4 mg/mL hyaluronidase (Type-S; Sigma, St Louis, USA) and 25 μM CaCl _2_-containing modified Tyrode’s solution for 7 to 12 min. Then, the left ventricle was minced into small pieces with the same solution containing 0.25 mg/mL collagenase, 2 mg/dL trypsin and 50 μM CaCl _2_ and incubated for 10 min for further digestion. Isolated cells were maintained at 37°C in a 5% CO _2_, 95% atmosphere and used for experiments within 6 h after dissociation.

### Loading of Fluo-3-AM or sodium Green-AM for fluorescence microscopy

Intracellular free Ca ^2+^ ([Ca ^2+^]i) and Na ^+^ ([Na ^+^]i) concentrations in single ventricular heart cells were measured with the calcium and sodium-sensitive fluorescent probes Fluo-3 and sodium Green-AM (Molecular Probes, Oregon, USA) respectively [ 25, 26]. Cells were loaded according to the method described previously [27]. Frozen stocks of Fluo 3-AM or sodium Green-AM were reconstituted in DMSO and diluted to a final concentration of 13.5 μM in Tyrode’s-BSA. Pluronic acid was added to the initial Sodium Green preparation to facilitate cell loading. Myocytes were attached to laminin-coated glass coverslips and then incubated for 45 min at room temperature, washed for 40 min, and further incubated for 15 min at room temperature to complete the hydrolysis of acetoxymethyl ester groups. The intensity of the fluorescence increases with an increase in [Ca ^2+^]i and [Na ^+^]i. Fluorescence intensity in the entire cell volume was detected, and average calcium or sodium fluorescence intensity values were calculated for each group. Free [Ca ^2+^]i and [Na ^+^]i are represented as the mean fluorescence intensity values relative to normal levels.

### Histopathologic analysis

Coronal sections of heart samples were fixed in 4% paraformaldehyde for 24‒48 h, dehydrated, and embedded in paraffin. Sections (4 μm thick) were cut and stained with hematoxylin and eosin (H&E) as described previously [28]. Masson’s Trichrome staining was performed using a reagent kit from Solarbio (Beijing, China) as described previously [29]. The area of myocardial fibrosis was quantified using a colored high-definition pathological image-text analysis system (HPIAS-1000; Champion Image Technology Corporation Limited, Wuhan, China). The ratio of fibrosis area to total myocardial area represents the degree of myocardial fibrosis.

### Gomori silver staining

Reticulum fiber staining was performed according to the Gomori method (Gomori 1947) using a Gomori silver impregnation staining kit (G1800; Solarbio Science & Technology, Beijing, China). Briefly, the stationary process, paraffin embedding and dehydration procedure were the same as described previously [28]. Subsequently, the oxidizing agent Gomori was added to the samples and incubated for 5 min at room temperature, and the samples were washed with running water for 30 s. Then, the sections were rinsed with the following solutions: 2.5% oxalic acid (2 min), 5% iron alum (5 min), and silver ammonia solution (3 min). The reducing agent Gomori was added to the samples and incubated for 1 min, and washed with running water for 10 min at room temperature. Then, the sections were dehydrated through a graded series of ethanol, cleared in xylene, and mounted with neutral gum.

### Electron microscopy and quantitative analysis

The myocardial mitochondrial morphology was observed by electron microscopy. In brief, hearts from all groups were cut into small 1-mm-thick pieces and immediately fixed in 2.5% glutaraldehyde. Then, ultrathin sections were examined and imaged with a JEM-1400plus transmission electron microscope (Electron Optics Laboratory, Tokyo, Japan). The mitochondrial area and aspect ratio (the ratio of length/width) were quantified by ImageJ (NIH, Bethesda, USA). According to its volume ratio in three-dimensional space, the relevant secondary parameters, volume density (Vv), shape factor (PE), average area (S) and average perimeter (L), were calculated.

### Construction and analysis of the Pue-HF-common target protein network

Network pharmacological analysis of the core target of Pue therapy for HF was performed using the method reported previously [30]. Briefly, TCM ( http://bionet.ncpsb.org/batman-tcp/), SwissTarget BATMAN-Prediction ( http://www.swisstargetprediction.ch/) and GeneCards database ( https://www.genecards.org/) were used to obtain the target of puerarin and treatment of heart failure respectively, and then the targets of Puerarin consociation sibeline targets for the treatment of heart failure were obtained in the VENNY platform ( https://bioinfogp.cnb.csic.es/tools/venny/). The processed Pue was then molecularly docked to NHE1 using AutoDock Tools ( https://autodock.scripps.edu/).

### RT-qPCR

Tissues were homogenized in TRIzol Reagent (Thermo Fisher Scientific, Waltham, USA), and total RNA was extracted according to the manufacturer’s instructions. The RNA concentration was determined using a NanoDrop 2000 spectrophotometer (Thermo Fisher Scientific). RNA was reverse-transcribed using the PrimeScript RT-PCR Kit (RR037A; TaKaRa, Shiga, Japan) according to the manufacturer’s protocol. PCR was performed using SYBR green qPCR mix (#CW0957M; CoWin Biosciences, Taizhou, China) on a StepOne Real-Time PCR System (Thermo Fisher Scientific). Ct values were obtained and normalized to the levels of mRNA of *GAPDH*, and then the relative level of mRNA expression was calculated using the 2 ^‒ΔΔCt^ method. The primers used are listed in Table 1.

**Table:** **Table 1** The sequences of the primers used for RT-qPCR in this study

Gene	Forward primer (5′→3′)	Reverse primer (5′→3′)
*IL-1β*	AATCTCACAGCAGCATCTCGACAAG	TCCACGGGCAAGACATAGGTAGC
*TNF-α*	ATGGGCTCCCTCTCATCAGTTCC	CCTCCGCTTGGTGGTTTGCTAC
*IL-6*	ACTTCCAGCCAGTTGCCTTCTTG	TGGTCTGTTGTGGGTGGTATCCTC
*TGF-β*	GACCGCAACAACGCAATCTATGAC	CTGGCACTGCTTCCCGAATGTC
*GAPDH*	GACATGCCGCCTGGAGAAAC	AGCCCAGGATGCCCTTTAGT

### Western blot analysis

Western blot analysis was performed as described previously [31]. Briefly, the total proteins were boiled in SDS sample buffer for 10 min. Proteins were separated by 10% SDS-PAGE and were electrically transferred to polyvinylidene difluoride membranes. Membranes were blocked for 2 h at room temperature in TBS containing 5% nonfat milk and then incubated with specific primary antibodies at 4°C overnight on an orbital shaker. Membranes were then washed and incubated with horseradish peroxidase (HRP)-conjugated secondary antibodies for 1 h at room temperature. Immunoreactive protein bands were visualized using the enhanced chemiluminescence (ECL) substrate and images were captured using a gel imaging system (Bio-Rad, Hercules, USA). Band densitometry analysis was performed with ImageJ.

### Immunohistochemistry (IHC)

IHC was performed using conventional methods as previously described [32]. Briefly, antigen retrieval was conducted using 10 mM sodium citrate buffer (pH 6.0) in a microwave oven for 15 min at 98°C, and endogenous peroxidase activity was quenched using 3% hydrogen peroxide (37°C for 10 min). After being blocked with normal horse serum, the sections were incubated with primary antibody at 4°C overnight, followed by incubation with horseradish peroxidase-conjugated secondary antibody at room temperature for 60 min. The slides were then processed with a 3,3′-diaminobenzidine (DAB) substrate kit (#3400; Thermo Fisher Scientific). Morphometric analyses were performed using ImageJ software in an operator-blind manner. For each sample, at least 5 randomly selected fields were analyzed, and the data were averaged.

### Statistical analysis

Data are expressed as the mean±standard error of the mean (SEM). *n* represents the number of independent samples but not technical replicates. Multiple comparisons were analyzed using one-way analysis of variance (ANOVA) followed by post hoc Tukey’s test. Data without a Gaussian distribution were log transformed before statistical analysis. Statistical analysis was performed using Prism software (GraphPad, San Diego, USA). All tests were run as two-tailed tests. *P*<0.05 was considered statistically significant.

## Results

### Pue attenuates myocardial injury and myocardial fibrosis in ADR-induced HF rats

**Figure 1:**
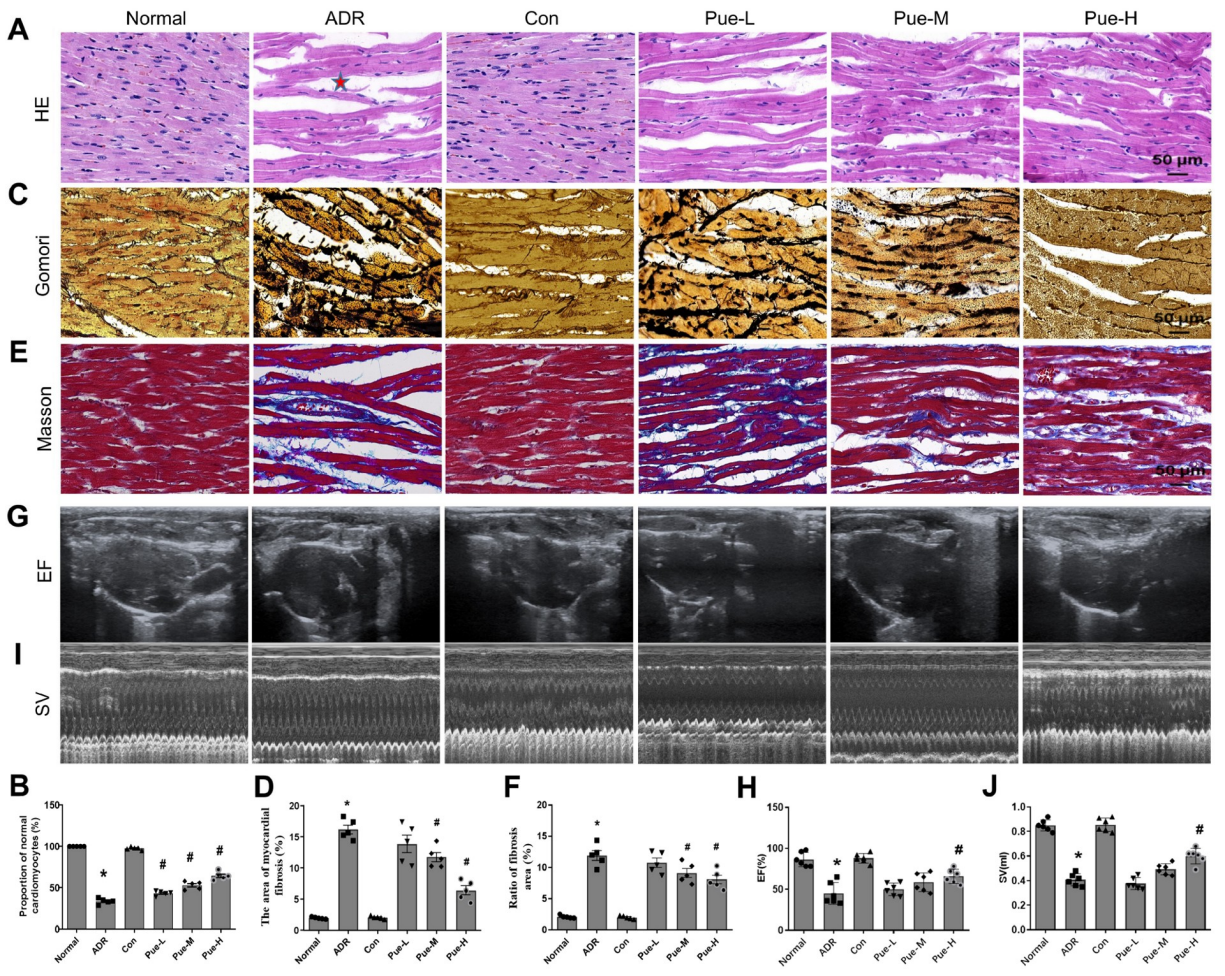
Figure 1 Pue prevented the progression of myocardial fibrosis and improved left ventricular function in ADR-induced HF rats (A) HE staining. (B) Normal cardiomyocyte ratio of HE-stained cells. (C) Gomori silver staining. (D) Quantitative analysis of the reticular fiber area in the myocardial tissue of each group. (E) Masson staining. (F) Quantitative analysis of collagen fiber area in each group. (G,I) Representative two-dimensional and M-mode short-axis echocardiographic images of left ventricular function. (H,J) Quantitative analysis of echocardiography parameters (EF% and SV) in each group. Data were analyzed by using one-way ANOVA. All data are expressed as the mean±SEM (n=5 for B,D,F; n=6 for panels H,J). *P<0.05 vs the Con group; #P<0.05 vs the ADR group.

The major cardiac structural remodeling of HF includes cardiac hypertrophy, fibrosis, inflammatory cell infiltration and edema, and even myocardial necrosis [33]. Myocardial fiber disruption and increased collagen with interstitial edema were observed in rat cardiomyocytes after 6 weeks of ADR induction. Pue treatment attenuated myocardial fiber breaks and edema, which was particularly significant in the high-dose group ( Figure 1A,B).

The content of reticular fibers ( Figure 1C,D) and collagen fibers ( Figure 1E,F) in ADR-induced rat myocardium was significantly increased, while treatment with middle- and high-dose of Pue reduced the expressions of collagen fibers and reticular fibers in myocardium and significantly attenuated the degree of myocardial fibrosis.

### Pue improves LV systolic function in rats with HF

Next, we measured the EF and SV (both reliable indicators of LV systolic function) by ultrasonography to evaluate the LV systolic function in each group of rats. Both myocardial EF ( Figure 1G,H) and SV ( Figure 1I,J) were significantly decreased in ADR-induced rats, indicating that ADR induction not only damages the myocardium structure but also causes ventricular systolic dysfunction. In contrast, treatment with high-dose Pue greatly increased the EF, resulting in an increase in SV, suggesting that Pue improves the contractile function of the heart (at least the LV).

### Pue regulates sodium and calcium concentration homeostasis

**Figure 2:**
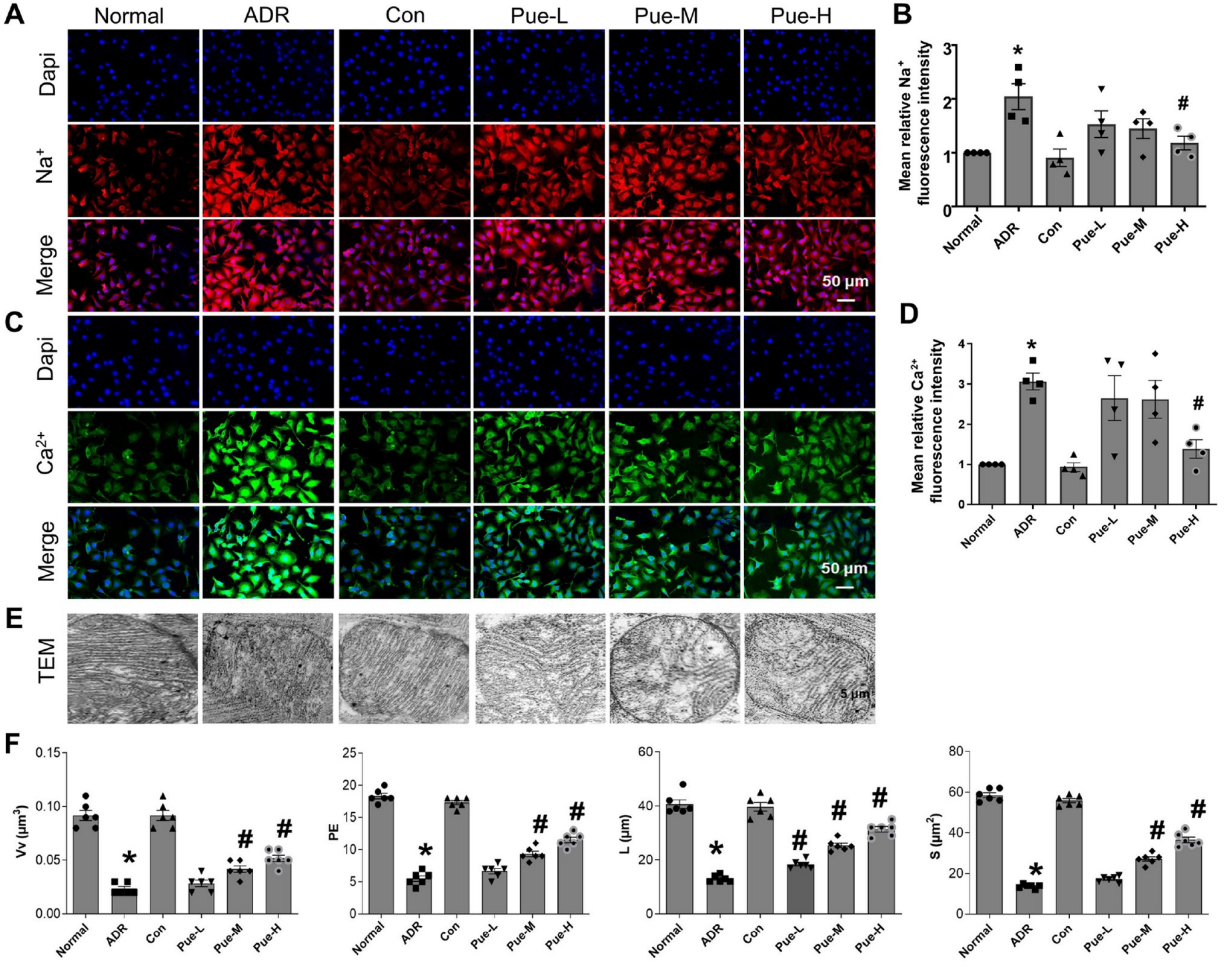
Figure 2 Pue reduced intracellular free sodium and calcium ion concentrations and attenuated mitochondrial damage caused by calcium overload Representative immunofluorescence images of [Na+]i (A) and [Ca2+]i (C) in single ventricular heart cells of each group. (B) Quantitation of cytoplasmic Na+ fluorescence. (D) Quantitation of cytoplasmic Ca2+ fluorescence. (E) Mitochondrial transmission electron microscopy. (F) Quantitative analysis of mitochondrial structure. Data are presented as the mean±SEM. *P<0.05 vs the Con group; #P<0.05 vs the ADR group, one-way ANOVA (n=4 for panels A,C; n=6 for E).

Whether ADR induction causes an imbalance in intracellular sodium and calcium ion concentrations and whether Pue can regulate intracellular sodium and calcium concentrations are our next concerns. The results showed that ADR induced an increase in intracellular free sodium ( Figure 2A,B) and calcium ( Figure 2C,D) ion concentrations in rat myocardium with significant differences, while treatment with high-dose of Pue significantly reduced intracellular free sodium and calcium ion concentrations and maintained intracellular ion concentration homeostasis.

### Pue alleviates mitochondrial damage in ADR-induced HF rats

By mitochondrial electron microscopy ( Figure 2E), mitochondrial swelling and striatum rupture were observed in the ADR group, which could be reversed by Pue treatment. Quantitative analysis of mitochondrial structure showed that Vv, PE, S and L were significantly increased in the middle- and high-dose Pue groups compared with the ADR group ( Figure 2F).

### Pue reduces NHE1 protein expression

**Figure 3:**
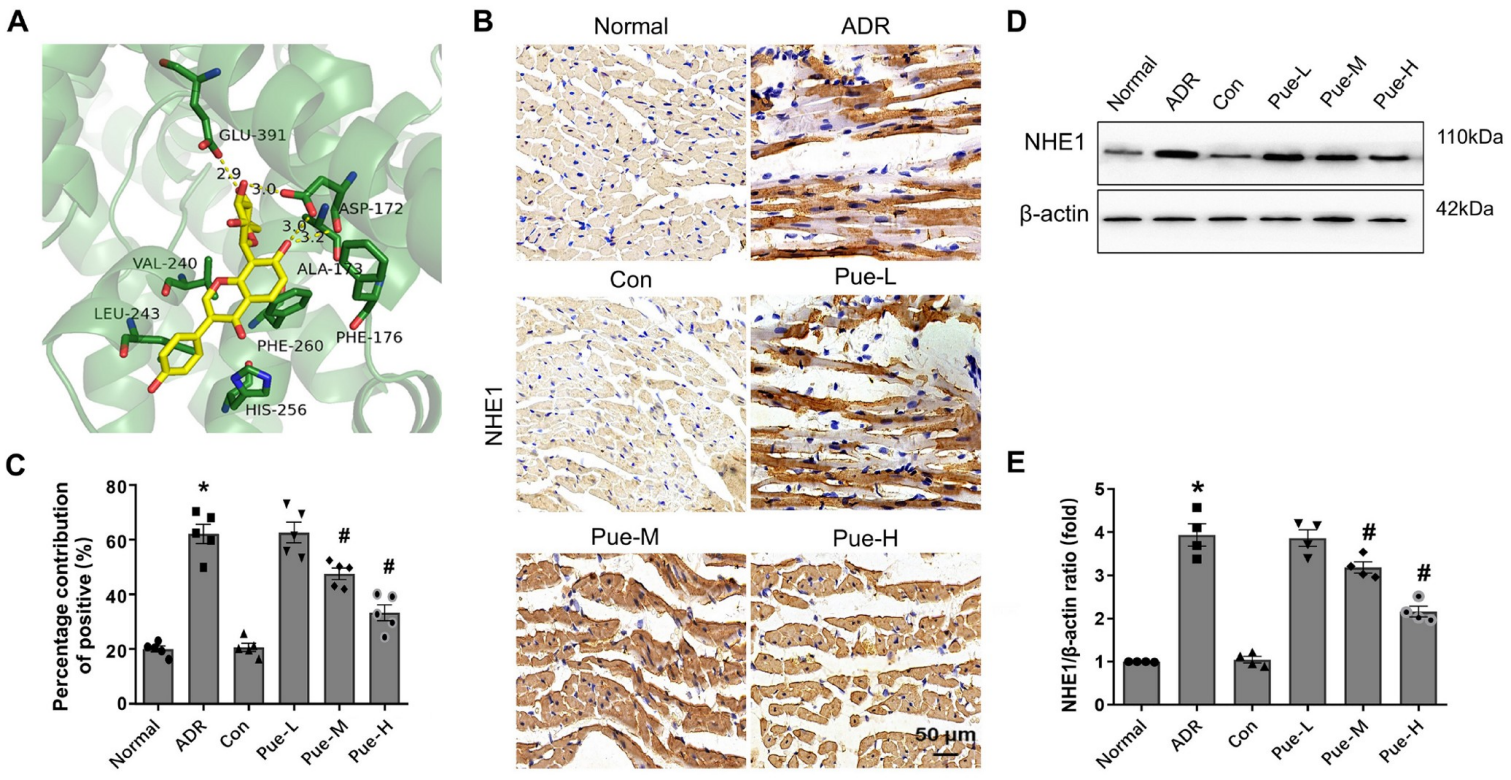
Figure 3 Effects of Pue on NHE1 expression in myocardial tissue of ADR-induced HF rats (A) Overall effect diagram of Pue and NHE 1 docking. (B) Immunohistochemistry images of NHE1 in myocardial tissues of each group. (C) Quantitative data of the positive expression of NHE1. (D) Representative western blots of NHE1 expression. (E) Statistical analysis of the protein expression of NHE1. Data are presented as the mean±SEM. *P<0.05 vs the Con group; #P<0.05 vs the ADR group, one-way ANOVA (n=5 for panel B; n=4 for D).

In the heart, hyperactivation of NHE1 has been linked to the development of different pathologies, such as myocardial fibrosis and HF [34]. However, the effect of Pue on NHE1 in the myocardium has not been reported. The molecular docking results showed that the binding energy of Puerarin-NHE1 was ‒8.0 kcal/mol, indicating good binding between Puerarin and NHE1 ( Figure 3A). To explore whether the protective effect of Pue against HF is related to NHE1, we examined the expression of NHE1 in the myocardium of each group by immunohistochemistry and western blot analysis. In ADR-induced rat myocardium, both immunohistochemical staining ( Figure 3B) and western blot analysis ( Figure 3D) results showed that ADR activated NHE1 in the myocardium, while medium and high doses of Pue reduced the activation of NHE1 ( *P*<0.05). These results suggested that Pue may exert myocardial protective effects by inhibiting the excessive activation of NHE1 and affecting the intracellular sodium ion concentration in the myocardium.

## Pue decreases the expression levels of relevant cytokines in a rat HF model

**Figure 4:**
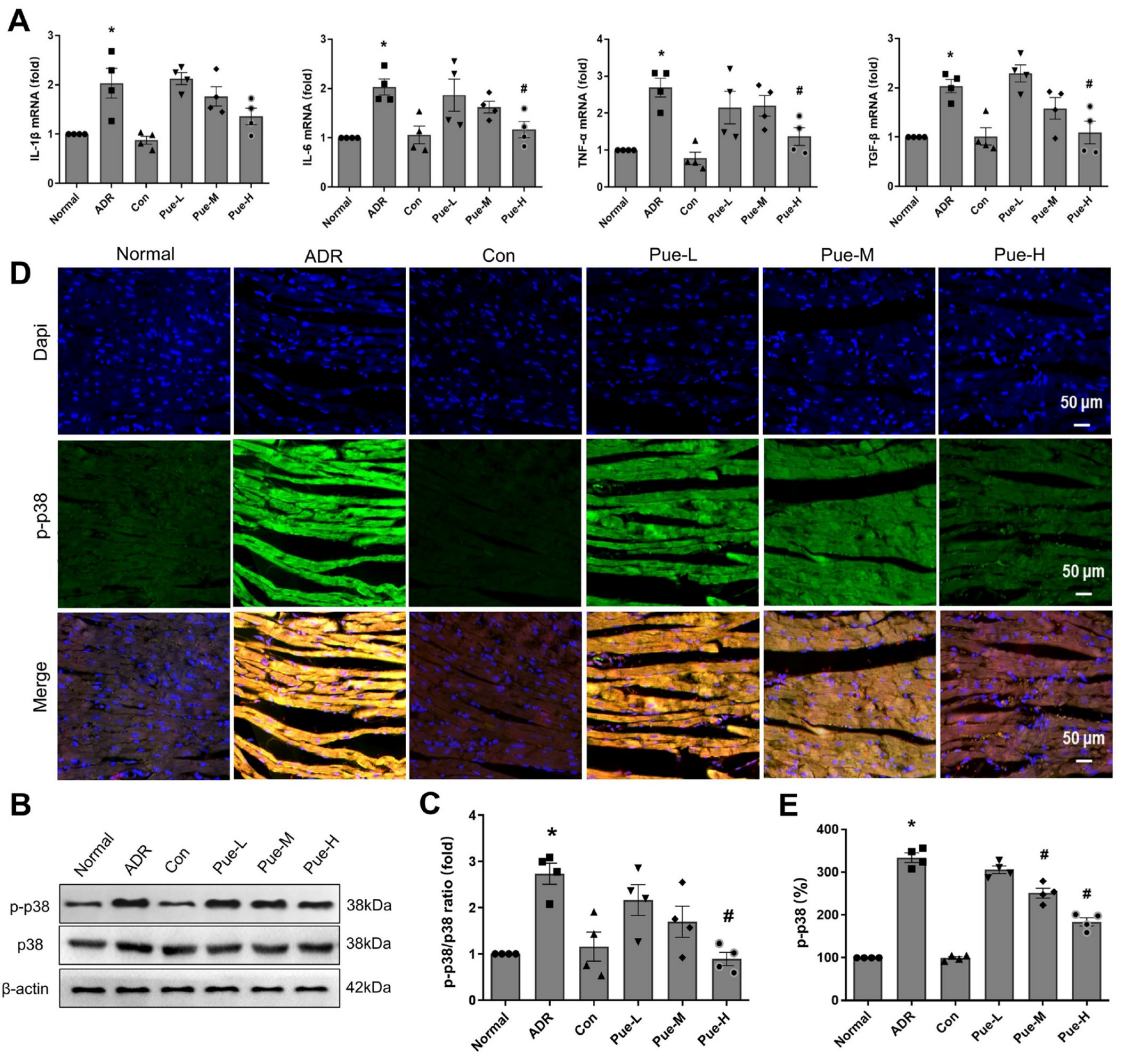
Figure 4 Pue inhibited p38 activation and reduced cardiac fibrosis and proinflammatory cytokine production in the myocardial tissue of ADR-induced HF rats (A) The mRNA levels of IL-Iβ, IL-6, TNF-αand TGF-β in the myocardial tissue of each group. (B) Representative western blots of p-p38 expression. (C) Statistical analysis of the ratio of p-p38/p38. (D) Immunofluorescence staining of p-p38 in myocardial tissues of each group. (E) Quantitative analysis of p-p38. Data are presented as the mean±SEM. *P<0.05 vs the Con group; #P<0.05 vs the ADR group, one-way ANOVA (n=4).

TGF-β is an important fibrogenic cytokine [35], and IL-1β, IL-6, and TNF-α [ 36– 38] are the major proinflammatory markers in the fibrotic response of the heart in mammalian cells. The qPCR results showed that TGF-β, TNF‑α and IL-6 mRNA levels were significantly upregulated in the ADR-induced myocardium of rats, while treatment with high-dose of Pue decreased the mRNA expression of these cytokines ( Figure 4A). These results indicate that Pue treatment can inhibit the progression of ADR-induced myocardial fibrosis and has significant anti-inflammatory effects, which may be one of the possible mechanisms by which Pue attenuates myocardial fibrosis in rats with HF.

### Pue inhibits the p38 pathway in ADR-induced HF rats

Total p38 protein expression and the phosphorylation level of p38 in the myocardium of each group were measured separately. As shown in Figure 4B,D, the phosphorylation level of p38 was elevated significantly in ADR-induced rat myocardium, and treatment with high-dose of Pue significantly attenuated the phosphorylation of p38 and inhibited the activation of p38.

## Discussion

In this study, we found that Pue significantly improved myocardial fibrosis and inflammatory infiltration and improved cardiac function in HF rats by establishing an ADR-induced HF model. Mechanistically, we found for the first time that Pue inhibited the overactivated NHE1 in HF, which may alleviate mitochondrial damage caused by calcium overload and improve HF by reducing the concentration of Na ^+^ and Ca ^2+^ ions; on the other hand, Pue inhibited the activation of the p38 pathway in HF, reduced the expression of TGF-β, and inhibited myocardial fibrosis.

Inflammation plays an important role in the pathophysiological process of HF. These proinflammatory cytokines can stimulate the release of many other inflammatory cytokines and transcription factors, activating immune cell transdifferentiation to proinflammatory and profibrotic subpopulations, which may promote myocardial hypertrophy and fibrosis [ 39– 41]. The mRNA level of proinflammatory cytokines in the myocardium was significantly reduced after 4 weeks of continuous treatment with Pue (150 mg/kg) ( Figure 4A), and myocardial pathological examination also showed reduced myocardial fiber disruption and alleviated interstitial edema. These results confirmed that Pue significantly attenuated the inflammatory response in the myocardium of ADR-induced HF rats.

In this study, we specifically labelled collagen fibers and reticular fibers in rat myocardium. We found that Pue treatment attenuated ADR-induced collagen ( Figure 1E) and reticular fiber content in rat myocardium ( Figure 1C), inhibited the formation and development of fibrosis, increased myocardial LV EF and SV ( Figure 1G and 1I), and greatly improved myocardial function. In cardiac tissue, activation of the TGF-β signaling pathway is associated with the development of cardiomyocyte hypertrophy and HF [ 42, 43]. Additionally, microvascular inflammation stimulates the secretion of TGF-β by monocyte-derived macrophages, which induces cardiac fibroblast proliferation and stimulates the phenotypic transformation of cardiac fibroblasts to myofibroblasts as well as extracellular matrix production. Myofibroblasts deposit collagen, and the increase in collagen may lead to fibrosis [44]. Moreover, TGF-β may simultaneously block matrix degradation by decreasing protease synthesis and increasing the levels of protease inhibitors [45]. We examined the expression of TGF-β in the myocardium of each group and found that Pue decreased the expression of TGF-β in ADR-induced myocardium, suggesting that the effect of Pue in reducing myocardial fibrosis may be related to the inhibition of TGF-β expression in myocardial tissue.

Some studies reported that Na ^+^ and Ca ^2+^ are closely associated with HF [46]. Different studies have also confirmed that Pue can exert myocardial protective effects by regulating Na ^+^ and Ca ^2+^ ion concentrations in cardiomyocytes [6]. We confirmed that ADR induced an increase in intracellular free Na ^+^ and Ca ^2+^ ion concentrations in rat myocardium and that treatment with high-dose of Pue indeed significantly reduced intracellular free Na ^+^ and Ca ^2+^ ion concentrations ( Figure 2A–D), but the mechanism is not fully understood.

NHE1 plays an important role in maintaining Na ^+^ and Ca ^2+^ ion concentrations in the myocardium [15]. More importantly, exacerbated NHE1 activity has been associated with pathological cardiac processes [47]. Activation of NHE1 allows intracellular Na ^+^ to accumulate and a consequent increase in Ca ^2+^ transient amplitude through reverse Na ^+^/Ca ^2+^ exchange with subsequent activation of deleterious pathways, including the calcium/calmodulin-dependent protein kinase-histone deacetylase signaling pathway and Ca ^2+^-dependent prohypertrophic signaling molecules [48], inducing increased cross-sectional area of cardiomyocytes, interstitial fibrosis, and decreased cardiac function. Dysregulation of Ca ^2+^ homeostasis is a hallmark of HF, and the resultant Ca ^2+^ overload contributes to mitochondrial dysfunction [49]. Our results revealed for the first time that Pue could affect NHE1 expression and inhibit ADR-induced NHE1 activation in an experimental HF model ( Figure 3B,D). We hypothesized that inhibition of NHE1 overactivation, reduction of [Na ^+^]i accumulation, and prevention of Ca ^2+^ overload and the resulting mitochondrial dysfunction may be potential cardioprotective mechanisms of Pue. In fact, NHE exchanger inhibitors have been proven to protect the heart against I/R injury [ 50, 51] and inhibit the development of myocardial hypertrophy and HF.

Different kinases are associated with the regulation of NHE1. It has been found that p38MAPK can regulate NHE1 activity [52] and plays an important role in the regulation of cardiac remodeling and cardiac contractility [ 53– 55]. Consistently, our results suggest that p38 is activated in ADR-induced rat myocardium and that Pue dose-dependently inhibits p38 activation. Most studies have shown that p38 activation promotes the development of HF due to extracellular matrix remodeling and cardiac fibrosis by activating the TGF-β signaling pathway [ 56– 58]. This is consistent with our results that Pue inhibits p38 activation and reduces cardiac fibrosis and proinflammatory cytokine production, suggesting that p38 blockade and subsequent inhibition of the TGF-β signaling pathway may be a possible mechanism by which Pue exerts its cardioprotective effects.

p38 also controls cardiomyocyte contractility, and p38 activation has been shown to have an anti-inotropic effect [ 59– 61]. Two main possibilities are proposed: altering intracellular pH and phosphorylating contractile proteins, thereby desensitizing cardiomyocytes to calcium, but the exact mechanism of inhibition of myofilament responsiveness to Ca ^2+^ is not clear. Our results offer the possibility that p38 activation may cause intracellular calcium overload via sodium-calcium exchange by causing enhanced NHE1 activity and thus decrease calcium sensitivity of myofilaments. Pue inhibits the activation of p38, which inhibits p38-mediated cardiac fibrosis and proinflammatory cytokine production, further improving the sensitivity of myocardium to calcium and enhancing myocardial contractility by inhibiting the activation of NHE1. This needs further validation.

Pue, as a vasodilator, dilates blood vessels and reduces myocardial oxygen consumption, which is the basic principle of its therapeutic effect on cardiovascular diseases. However, care should be taken in the choice of dosage to avoid adverse reactions such as hypotension.

In conclusion, Pue is an effective drug for the treatment of HF in the ADR-induced HF rat model. Our results suggest that Pue may exert myocardial protective effects by inhibiting the activation of p38MAPK and its downstream NHE1 activation, inhibiting myocardial fibrosis and the production of proinflammatory cytokines, attenuating mitochondrial damage caused by calcium overload, and improving myocardial contractile function. Nevertheless, this study mainly focused on the effects of Pue on HF and the potential mechanisms at the animal level, lacking validation by *in vitro* experiments. In addition, we did not further characterize the interaction of p38 with NHE1 and the effect of *NHE1* knockdown on the efficacy of Pue. We believe that it would be interesting and important to characterize the interaction of p38 with NHE1 *in vitro* as well as to observe the efficacy of Pue using *NHE1*-knockout animals in future studies.
